# Acute Rhabdomyolysis Associated with Coadministration of Levofloxacin and Simvastatin in a Patient with Normal Renal Function

**DOI:** 10.1155/2014/562929

**Published:** 2014-07-22

**Authors:** Maria Paparoupa, Sebastian Pietrzak, Adrian Gillissen

**Affiliations:** Department of Pulmonary Diseases, General Hospital Kassel, Kassel School of Medicine, Mönchebergstraße 41-43, 53125 Kassel, Germany

## Abstract

We report a rare case of severe acute rhabdomyolysis in association with coadministration of levofloxacin and simvastatin in a patient with normal renal function. A 70-year-old Caucasian male was treated due to community acquired pneumonia with levofloxacin in a dosage of 500 mg once and then twice a day. On the 8th day of hospitalization the patient presented with acute severe rhabdomyolysis requiring an intensive care support. After discontinuation of levofloxacin and concomitant medication with simvastatin 80 mg/day, clinical and laboratory effects were totally reversible. Up to now, levofloxacin has been reported to induce rhabdomyolysis mainly in patients with impaired renal function, as the medication has a predominant renal elimination. In our case renal function remained normal during the severe clinical course. According to a recent case report rhabdomyolysis was observed due to interaction of simvastatin and ciprofloxacin. To our best knowledge this is the first case of interaction between simvastatin and levofloxacin to be reported. This case emphasizes the need of close monitoring of creatine kinase in patients under more than one potentially myotoxic medication especially when patients develop muscle weakness.

## 1. Introduction

Acute rhabdomyolysis is well-known as a clinical and laboratory syndrome associated with myoglobinuria, electrolyte disorders, and acute renal failure. Its diagnosis is based on the elevated creatine kinase levels after exclusion of myocardial infarction. Although initially described to be induced by crush injury or trauma, more common causes in hospitalized patients include coadministration of interacting drug agents [1]. The identification of a drug-induced rhabdomyolysis is important because the adverse effect is usually reversible after withdrawal of the suspected compound.

Particularly statins are well-known for their myotoxic potential especially in genetically susceptible individuals [2]. The symptoms of the myotoxic effect vary from simple myalgia to severe rhabdomyolysis [3]. Rhabdomyolysis caused by statins is mainly observed at the beginning of the treatment, after changing the agent [4], or even after increasing the dose of the subscribed medication [5]. The mean onset of myopathic side effects is approximately 6 months after initiation of treatment, although symptoms can occur at any time during the statin therapy [6].

Levofloxacin is a widely prescribed antibiotic for the treatment of community acquired pneumonia. It has a predominant renal elimination and dose reduction is necessary in patients with renal dysfunction [7]. Acute rhabdomyolysis due to Levofloxacin has been previously reported in elderly patients [8], in patients under hemodialysis [9], or after renal transplantation [10]. We report a rare case of severe acute rhabdomyolysis associated with coadministration of levofloxacin and simvastatin in a 70-year-old Caucasian male with normal renal function.

## 2. Case Presentation

A 70-year-old Caucasian male with previous medical history of coronary artery disease, hypertension, hyperlipidemia, and atrial fibrillation was presented with persistent dyspnea and cough for several days. An externally performed chest X-ray revealed consolidation of the upper lobe of the left lung. Having signs of community acquired pneumonia ampicillin/sulbactam was commenced and after developing diarrhea the antibiotic regime was changed to intravenous levofloxacin 500 mg/day. After 5 days of consecutive treatment respiratory symptoms remained unchanged and the patient was subsequently admitted to our institution for further diagnostic procedures.

At the day of admission a clinical work up revealed reduced general health but no further clinical abnormalities. He denied back and joint pain or skin rashes. Routine laboratory exams were performed and have shown elevated liver (aspartate aminotransferase [ASAT] 129 U/L, reference range 5–34 U/L and alanine aminotransferase [ALAT] 113 U/L, reference <55 U/L) and muscle enzymes (creatine kinase [CK] 2521 U/L, reference range 30–200 U/L) without signs of myocardial injury (quotient CKMB/CKMM 2). As the inflammatory markers were still significantly high (C-reactive protein [CRP] 182 mg/L, reference <5 mg/L) and the renal function was intact (blood potassium 4.10 mmol/L and creatinine concentrations 0.8 mg/dL, resp.) levofloxacin therapy has been continued in a higher dosage (500 mg/2 ∗ day). Serum brain natriuretic peptide was normal, ruling out cardiogenic dyspnea. Active medication including simvastatin 80 mg/day, metoprolol 25 mg/2 ∗ day, aspirin 100 mg/day, digitoxin 0,07 mg/day, and furosemide 40 mg/day was maintained. About 20 days before the admission of the patient to our department a former dose of simvastatin 40 mg/day was doubled to 80 mg/day as referred from his general physician. The reason for increasing the simvastatin dose was a persistent hypercholesterolemia despite the intake of the former antilipid therapy. 

On the fourth day of hospitalization (overall day 8 of levofloxacin therapy) the patient complained about progressively increased bilateral legs and arms weakness involving both distal and proximal muscle groups. He further reported difficulty to stand up from his bed, dress up, or even use his pen to write. There was no bowel or bladder incontinence or further neurological symptoms. Physical examination revealed diffuse extremity weakness but no other neurological findings or signs of dermatomyositis. At this time muscle enzymes ([CK] 31539 U/L) and parameters of liver toxicity ([ASAT] 486 U/L and [ALAT] 182 U/L) increased dramatically indicating a rhabdomyolysis and the Patient was immediately treated with intravenous crystalloid hypotonic solution (100 mL/h). We also proceded to discontinuation of simvastatin and levofloxacin, urine alkalinization and physical therapy.

A remarkable increase of muscle enzymes during the next days (maximum [CK] 159.450 U/L) and severe generalized weakness, including respiratory insufficiency, made the admission to the ICU necessary, for implementation of noninvasive ventilation. As the renal retention parameters remained normal no hemodialysis was performed. Wide range of serology markers excluded other causes of rhabdomyolysis. A computer tomography of the chest showed withdrawal of the infiltration, although a 2 cm large tumor on the upper left lobe remained. A transbronchial biopsy confirmed the presence of nonsmall cell lung cancer.

**Figure 1:**
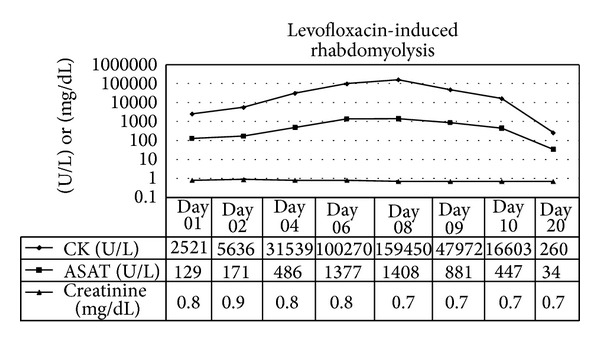
Serum creatinine, creatine kinase [CK], and aspartate aminotransferase [ASAT] during the severe clinical course. Notice the same curve morphology regarding the CK and ASAT evolution.

Within a few days his symptoms improved significantly and his muscle and liver enzymes normalized (Figure 1). The patient was discharged at the 26th day of hospitalization with the diagnosis of iatrogenic rhabdomyolysis with acute hepatocellular damage. He presented again to the department of radiotherapy for the treatment of his lung carcinoma and his laboratory parameters were totally normal. The previous dosage of simvastatin 40 mg/day was already resumed by his general physician.

## 3. Discussion

The diagnosis of acute severe rhabdomyolysis in our case is clear. The most probable cause for it is the treatment with levofloxacin, because there was a close temporal sequence between levofloxacin administration and the appearance of the symptoms, as well as clinical and laboratory recovery when the medication was discontinued. The adverse reaction appeared to be more severe when the dosage of levofloxacin was increased. Accordingly to Naranjo's probability scale a probable relationship between levofloxacin and rhabdomyolysis (total score 5) was confirmed even if simvastatin was considered to be an alternative cause of the adverse event (Table 1). Furthermore, all other potential reasons of muscle damage, such as* Influenza A and B virus* [11],* Parainfluenza type 1 and 2 virus* [12],* Coxsackie virus* [13],* HIV 1-2 virus* [14], endocrinological abnormalities [15], and autoimmune myositis could be serologically excluded. Serum and urinary antigen and antibodies exams excluded* Legionella pneumophila*,* Mycoplasma pneumoniae*,* Chlamydia pneumoniae*, and* Streptococcus pneumoniae* as potential pathogens of the community acquired pneumonia.

Levofloxacin has a predominant renal elimination and a dose reduction is necessary in patients with impaired renal function [7]. Acute rhabdomyolysis has been previously reported in patients under hemodialysis [9] or after renal transplantation [10]. Petitjeans et al. described in 2003 a case of an elderly patient with severe rhabdomyolysis, suffering from acute renal failure [8]. In our case renal function was normal at the beginning of the therapy and remained intact all along the severe clinical course, indicating that the adverse event is also probable in patients without renal impairment. An ofloxacin-induced rhabdomyolysis in previously healthy individuals has been reported from Hsiao et al. in 2005 [16], while other fluoroquinolones may also lead to this very rare adverse event mostly associated with acute kidney injury [17]. The histological effect of fluoroquinolones on the muscle cell has been demonstrated several years ago in juvenile rats, where no necrosis or lysis was seen, but the muscle fibers became atrophic [18].

The role of simvastatin in our case has to be also considered, as the medication has a very well confirmed myotoxicity especially when coadministrated with antibiotics, such as macrolides [19], fusidic acid [20], and azole antifungals [21]. According to a recent case report rhabdomyolysis was observed due to interaction of simvastatin and ciprofloxacin [22]. To the best of our knowledge, this is the first case of interaction between simvastatin and levofloxacin to be reported. Simvastatin is metabolized mainly in the liver by cytochrome CYP 3A4 and its active metabolite simvastatin acid is metabolized by cytochrome CYP 2C8 [23]. Levofloxacin is a weak inhibitor of cytochrome CYP 1A2 and has no effect on cytochromes CYP 3A4 and CYP 2C8. As approximately 80% of levofloxacin is eliminated as unchanged drug in the urine through glomerular filtration and tubular secretion and only minimal metabolism occurs with the formation of no active metabolites [24], most likely other mechanisms are involved in the drug interaction of our case. Statins are substrates and strong inhibitors of P-glycoprotein (P-gp), also known as MDR1, which is a cellular drug efflux-transporter, responsible for the bioavailability of lots of medications [25]. In vitro studies have shown that levofloxacin is a potent inhibitor of P-gp-mediated efflux system [26]. According to this knowledge, levofloxacin may have blocked the metabolism of simvastatin leading to increased toxicity.

## 4. Conclusion

Our case report highlights the importance of close monitoring in patients under more than one potentially myotoxic medication, especially when patients develop new muscle weakness, by checking the serum creatine kinase and performing liver function tests.

## Figures and Tables

**Table 1:** Naranjo's probability scale for our case.

Naranjo's algorithm questions	Answer	Score
(1) Are there previous conclusive reports of this reaction?	Yes	+1

(2) Did the adverse event appear when the drug was administered?	Yes	+2

(3) Did the adverse reaction improve when the drug was discontinued or a specific antagonist was administered?	Yes	+1

(4) Did the adverse reaction reappear when the drug was readministered?	Do not know	0

(5) Are there alternative causes (or other drugs) that could on their own explain the adverse reaction?	Yes	−1

(6) Did the adverse reaction reappear when a placebo was given?	No	+1

(7) Was the drug detected in the blood (or other fluids) in concentrations known to be toxic?	No	0

(8) Was the reaction more severe when the dose was increased or less severe when the dose was decreased?	Yes	+1

(9) Did the patient have a similar reaction to the same or similar drugs in any previous exposure?	Do not know	0

(10) Was the adverse event confirmed by any objective evidence?	No	0

	Total score:	5

The total score calculated from this table defines the category to which an adverse event belongs to.

The categories are defined as shown below:

definite (certain): >8;

probable: total score 5–8;

possible: total score 1–4;

doubtful (unlikely): total score <1.
